# RNA gene profile variation in peripheral blood mononuclear cells from rhesus macaques immunized with Hib conjugate vaccine, Hib capsular polysaccharide and TT carrier protein

**DOI:** 10.1186/s12865-018-0240-5

**Published:** 2018-01-25

**Authors:** Jing Tang, Ying Zhang, Xiaolong Zhang, Yun Liao, Yongrong Wang, Shengjie Ouyang, Yanchun Che, Miao Xu, Jing Pu, Qi Shen, Zhanlong He, Qiang Ye, Qihan Li

**Affiliations:** 1 Yunnan Key Laboratory of Vaccine Research and Development on Severe Infectious Diseases, Institute of Medical Biology, Chinese Academy of Medical Sciences and Peking Union Medical College, No. 935 Jiaoling Road, Kunming, Yunnan 650118 China; 2 National Institutes for Food and Drug Control, Beijing, China

**Keywords:** *Haemophilus influenzae* type b (Hib), Conjugate vaccine, Gene profile

## Abstract

**Background:**

The *Haemophilus influenzae* type b (Hib) conjugate vaccine has been widely used in children to prevent invasive Hib disease because of its strong immunogenicity and antibody response induction relative to the capsular polysaccharide (CPS) antigen. The data from vaccine studies suggest that the conjugate vaccine contains carrier proteins that enhance and/or regulate the antigen’s immunogenicity, but the mechanism of this enhancement remains unclear.

**Methods:**

To explore the immunological role of the conjugate vaccine, we compared the immune responses and gene profiles of rhesus macaques after immunization with CPS, carrier protein tetanus toxoid (TT) or conjugate vaccine.

**Results:**

A distinct immune response was induced by the Hib conjugate vaccine but not by CPS or carrier protein TT. The genes that were dynamically regulated in conjunction with the macaque immune responses to the conjugate vaccine were investigated.

**Conclusions:**

We propose that these genes are involved in the induction of specific immunity that is characterized by the appearance and maintenance of antibodies against Hib.

**Electronic supplementary material:**

The online version of this article (10.1186/s12865-018-0240-5) contains supplementary material, which is available to authorized users.

## Background

*Haemophilus influenzae* type b (Hib) is a widely recognized member of the *Haemophilus* genus that directly causes respiratory infectious disease with characteristic manifestations of tympanitis, bronchitis and pneumonia in children of all ages, particularly in infants and 1-year-olds [1–3]. Notably, this pathogen is associated with purulent meningitis in a specific ratio in all Hib-infected patients [1, 4]. Epidemiological studies of this pathogen have primarily been performed in developed countries and have shown that the incidence in children under the age of 5 in areas such as the US, France, and Switzerland is approximately 20–100/100,000 [5–9], but few data have been reported for developing areas. Although no systematic, epidemiological study of Hib has been performed in mainland China, studies of this pathogen in a small number of populations in different regions of China have indicated that the potential incidence of pediatric infections in mainland China is noteworthy [10, 11]. Numerous studies have addressed Hib structure and immunology [12, 13], leading to the licensure of a preventive vaccine against Hib infection in the 1980s [1]. Based on the results of a structural study of the Hib agent that suggested that the bacterium presented a capsular polysaccharide (CPS) antigen and thallus antigen, the early vaccine was prepared with polyribosylribitol phosphate (PRP), which is composed of ribosylribitol phosphate as the basic unit [14, 15]. The CPS antigen is the primary component of the 1st generation Hib vaccine and has been widely used to vaccinate children. Clinical observation of the use of this vaccine in pediatric populations indicated that it induced a remarkable immune response in children 18 months of age or older but did not provoke a satisfactory response in children younger than 18 months [16–19]. Subsequent Hib conjugate vaccines were developed based on a CPS antigen-binding protein (i.e., diphtheria toxoid (DT), tetanus toxoid (TT) and the *N. meningitidies* outer membrane protein (OMP) Hib thallus antigen protein) [18, 20]. Previous studies of this conjugate vaccine in mice and macaques have shown that remarkable immunity is induced by immunization with this vaccine compared to that induced by the CPS vaccine; the immunity usually presents as an increased antibody response in serum [21]. The results of additional clinical trials comparing conjugate Hib vaccines produced with various carrier proteins, including DT, TT and OMP, suggested that one or two immunizations induced a lower antibody response in children of various ages than three immunizations [22, 23]. The data indicated that the antibody levels of Hib vaccines conjugated with DT, TT, and OMP were 0.06, 0.05, and 0.83 μg/ml, respectively, after the first inoculation and that the levels increased to 0.14, 0.26, and 1.22 μg/ml after the second inoculation [23]. However, the levels increased further to 0.28, 3.64, and 1.14 μg/ml after the third inoculation [23]. Based on these data, a routine immunization schedule for the Hib conjugate vaccine has been recommended for children worldwide by the WHO [24], and the vaccines have been used extensively in multiple countries, including China [25, 26]. The increased immunogenicity associated with the Hib conjugate vaccine compared with that of the CPS vaccine suggests that binding of the semi-antigen CPS to the carrier protein would provide an effective antigen for immunization of individuals [27–30].

In previous studies, the immunogenicity of Hib CPS antigen and its protein conjugates were studied in juvenile and infant rhesus macaques [31, 32], which suggest that the conjugate of CPS and TT, DT and OMP are capable of inducing stronger specific antibody responses than CPS alone in this animal model [31–33]. Although those studies indicated unequal level of antibody induced against various conjugate vaccine including Hib-TT, Hib-CT and Hib OMPC and confirmed their immunogenicity, but also suggested the role of these carrier protein in the immunogenicity of Hib conjugate vaccine in human should be studied further [33, 34]. To better understand the immunologic mechanism of this conjugate vaccine, especially the role of carrier protein to enhance immunogenicity of CPS via the immunization schedule of three doses, we conducted a study using rhesus macaques, which are closely genetically related to humans, in which we assessed the mRNA profiles of rhesus macaque peripheral blood mononuclear cells (PBMCs) after immunization of the animals with the Hib conjugate vaccine, the Hib polysaccharide antigen or the carrier protein TT. Using comparison and analysis of these mRNA gene profiles, we investigated the molecular mechanism of immunity induced by the Hib conjugate vaccine and identified several genes that are likely to play a role in specific and effective immune responses against Hib in rhesus macaques. These genes might be induced by CPS antigen and carrier protein and might play the roles involved in the enhancement of immunogenicity of Hib conjugate vaccine.

## Methods

### Animals and ethics

The animal experiments were designed according to the principles of the Guide for the Care and Use of Laboratory Animals [35] and Guidance for Experimental Animal Welfare and Ethical Treatment [36]. The protocols were reviewed and approved by the Experimental Animal Ethics Committee of the Institute of Medical Biology, Chinese Academy of Medical Sciences for animal welfare (Approval number: YISHENGLUNZI [2015] 12).

Rhesus monkeys were separately bred in cages and fed according to the guidelines of the Committee on Experimental Animals at the Institute of Medical Biology, Chinese Academy of Medical Sciences. The housing conditions, experimental procedures and animal welfare measures were in accordance with the local laws and guidelines for the use of laboratory non-human primates and were compliant with the recommendations of the Weatherall report [35]. The monkeys were maintained under the care of veterinarians. The animals were bred in separate cages in a large room under BSL-2 conditions with sufficient fresh air and natural light; the housing conditions permitted the animals to have visual, olfactory and auditory interactions with other monkeys. The temperature of the room was maintained at approximately 25 °C during the experiments. Food and water were readily available to the animals, and appropriate treats and vitamins were provided. The animals were given access to environmental enrichment, including approved toys, to promote their psychological well-being. None of the animals were sacrificed as part of the experiment. No abnormalities were noted in any of the animals for the 6 months following the study; during this time, the animals were returned to the colony and remained under the care of a veterinarian.

### Antigens used for immunization

The *Haemophilus influenzae* type b conjugate vaccine was purchased from Beijing Zhifei Lvzhu Biopharmaceutical Co., Ltd. (Beijing, China; 10 μg *H. influenza* type b polysaccharide conjugated to 30 μg TT per 0.5 ml). Pure TT and polysaccharide were used as the control antigen (Beijing Zhifei Lvzhu Biopharmaceutical Co., Ltd., Beijing, China).

### Vaccination of monkeys

Juvenile rhesus monkeys (Six- to eight-month-old; 1.2 ± 0.5 kg) were randomly immunized in groups of three with the CPS antigen, carrier protein TT or conjugate vaccine via intramuscular injection (i.m.) in the anterolateral thigh on days 0, 30 and 60 using the formulations and immunization doses shown in Additional file 1: Table S1. The monkeys were bled on days 0, 30, 60 and 90. Sera and PBMCs were collected for ELISA analyses and microarray assays, respectively.

### ELISA analysis

The anti-PRP antibody concentration in the samples was measured using a standard ELISA protocol [37].Briefly, 96-well plates were coated with Ty-PRP (1:4000; National Institutes for Food and Drug Control, China) overnight. Before adding the sera, the wells were blocked at room temperature (RT). The horseradish peroxidase (HRP)-conjugated goat-anti-monkey IgG/IgA/IgM used as the secondary antibody (Sigma-Aldrich, Inc., St. Louis, MO, USA) was diluted 1:100,000. The substrate (Kinghawk Pharmaceutical Co., Ltd., Beijing, China) was incubated for 30 min at RT, and the reaction was terminated with the stop buffer, followed by absorbance measurements at 450 and 620 nm. With the reason that there was not available of standard anti-serum of macaque, the international standard human serum against Hib obtained from Sanofi-Aventis, was used as reference control. This standard serum was diluted in concentration of antibody in 10, 20, 40, 80, 160, 320 ng/ml and was as the reference for the evaluation of animal serum. The comparison of concentration of human antibody and titer of diluted macaque serum were determined in the light absorbance value in wave length of 450 nm (Additional file 1: Figure S1). The pre-immunization sera of macaques were used as the negative controls for this study. The goat-anti-monkey IgM was used as the secondary antibody (Sigma-Aldrich, Inc., St. Louis, MO, USA) to test the IgM titer induced by the conjugate, polysaccaharide antigen and the TT carrier. The carrier protein-specific IgG was evaluated using ELISA Assay kit for Tetanus Toxoid IgG (Vida-Bio Co. Ltd., Wuhan, China).

### RNA extraction

PBMCs were isolated from the whole blood by density gradient centrifugation over a Lymphoprep (Ficoll-Paque PREMIUM; GE Healthcare, Piscataway, NJ, USA). Total RNA from the PBMCs was isolated using the TRIzol Reagent (Invitrogen, CA, USA) and purified with the RNeasy Mini Kit (QIAGEN, GmBH, Germany). The RNA Integrity Number (RIN) was evaluated to inspect RNA integrity using an Agilent Bioanalyzer 2100 (Agilent, CA, USA). The extracted RNA was frozen in 95% ethanol until further use.

### Microarray assay

The Whole Rhesus Monkey Genome Microarray (G2519F-026806, Agilent, CA, USA) was chosen to screen for gene expression in monkey PBMCs. Gene Chip microarray experiments were conducted at the National Engineering Center for Biochip in Shanghai, China, according to the procedures in the Agilent technical manual. Briefly, the mRNA that was purified from total RNA after rRNA removal was amplified and transcribed into fluorescent cRNA using the Low Input Quick Amp Labeling protocol (Agilent).Labeled cRNA was purified using the RNeasy Mini Kit (QIAGEN, GmBH, Germany). Each slide was hybridized with 1.65 μl of Cy3-labeled cRNA using the Gene Expression Hybridization Kit (Agilent) in a hybridization oven (Agilent). After 17 h of hybridization, the slides were washed in staining dishes (Thermo, MA, USA) with the Gene Expression Wash Buffer Kit (Agilent). Slides were scanned using an Agilent Microarray Scanner (Agilent). The raw data were obtained using the Feature Extraction Software 10.7 (Agilent) and normalized using the quantile algorithm with Gene Spring 11.0 (Agilent). The systematic bioinformatic analyses of microarrays were processed by Novel Bioinformatics Co., Ltd. (Shanghai, China). Briefly, the normalization value was set to 1. The differentially expressed genes, with false discovery rate (FDR) values < 0.05 and Log_2_ (fold changes) ≥1.5, were analyzed compared to the samples collected at day 0. The significant pathway network (Gene-Act-Network) was determined from these differentially expressed genes using the Kyoto Encyclopedia of Genes and Genomes (KEGG) database, according to the relationship among the genes and proteins in the database [17, 38–40]. The raw microarray data were submitted to the Gene Expression Omnibus database and are available under the accession number GSE90481.

### Real-time RT-PCR

The gene expression levels were measured by real-time RT-PCR. The primers are listed in Additional file 1: Table S2. Briefly, total RNA was extracted from the PBMCs as described above. For quantification, a single-tube RT-PCR assay was performed using the 1-step RT-PCR Master Mix in a 7500 Fast Real-Time RT-PCR system (Applied Biosystems, Foster City, CA, USA). The following protocol was used for all PCR assays: 5 min at 42 °C and 10 s at 95 °C, followed by 40 cycles at 95 °C for 5 s and 60 °C for 30 s.

#### Flow cytometry

PBMCs were isolated by density gradient centrifugation with Lymphorprep medium (Ficoll-Paque PREMIUM; GE Healthcare, Piscataway, NJ, USA), and were counted by flow cytometry using anti-monkey CD3^+^ antibodies (BD Biosciences, San Diego, CA, USA), anti-CD69 antibodies (BD Biosciences) and anti-ITK antibodies (abcam, Cambridge, UK), according with the protocol as previous [41].

#### T cells proliferation and cytokine production

PBMCs from the rhesus macaques were used to measure T cell proliferation and cytokine secretion. PBMCs were placed in culture with CFSE (1.5 μM/well; BD Biosciences) and the stimulus (10 μg/ml TT) or positive control (LPS, 5 μg/ml) at 2 × 10^5^ cells/ml in RPMI 1640. Then, plates were incubated at 37 °C 5% CO_2_. After 36 h, the supernatants (50 μl) were obtained for cytokines analysis and cells were obtained for T cell proliferation by flow cytometry.

*--- cytokines analysis.* The cytokines productions were tested by NHP CBA analysis kit (BD Biosciences) according with the protocol [42].
--- *T cell proliferation.* The newly increased T cells were counted by flow cytometry co-marked with CD3^+^ antibodies and CSFE (BD Biosciences).

#### Statistical analysis

The non-parametric Kruskal-Wallis test was applied for comparisons between different groups using the GraphPad Prism software (San Diego, CA, USA). A *P*-value of < 0.05 was considered significant.

## Results

### A specific immune response was induced in Hib conjugate vaccine-immunized macaques but not in Hib CPS antigen- or carrier protein TT-immunized macaques

**Fig. 1:**
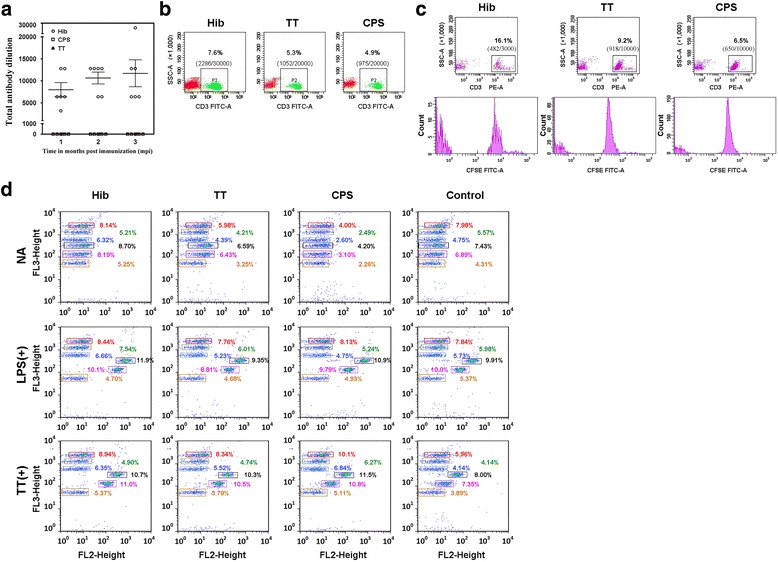
Specific immune responses after immunization with the Hib conjugate vaccine, the Hib capsular polysaccharide antigen or the carrier protein TT. **a** Specific antibody responses induced by Hib conjugate vaccine, CPS and TT. Antibody dilution (y-axis) was showed. Samples were obtained at 1, 2 and 3 months after the 1st immunization. **b**. The total number of T cells was counted following immunized with Hib conjugate vaccine, CPS and TT. The percent of T cells was shown above the rectangle, and the absolute numbers were shown in brackets. Samples were obtained at 1 month after complete course vaccination (3 months after the 1st immunization). Thirty thousand total cells were collected in Hib vaccine and 20,000 total cells were collected in CPS and TT group. **c**. TT induces the proliferation of T cells from the monkeys in Hib vaccine group. PBMCs were obtained at 1 month after complete course vaccination (3 months after the 1st immunization) and plated at 2 × 10^5^ cells/well. The percent of T cells was shown above the rectangle, and the absolute numbers were shown in brackets. Samples were obtained at 36 h after adding stimulate. Three thousand total cells were collected in Hib vaccine and 10,000 total cells were collected in CPS and TT group. **d**. TT induces the cytokines secretion of T cells from the monkeys in Hib vaccine group and. PBMCs were obtained at 1 month after complete course vaccination (3 months after the 1st immunization). Samples were obtained at 24 h after adding stimulate

Previous Hib conjugate vaccine trials in human subjects demonstrated good immunogenicity in all immunized pediatric groups after three inoculations, including in children of all ages, whereas the Hib polysaccharide vaccine elicited a lower antibody response in children over 18 months of age [16, 43]. However, a similar study in mice failed to observe age-based variations in immunogenicity [44]. In the present study of rhesus macaques, we selected 6- to 8-month-old animals with physiological features resembling those of 2- to 3-year-old humans [45, 46]. To identify effective immunization outcomes, the conjugate vaccine, the Hib polysaccharide antigen and the TT carrier were used to immunize the macaques according to the routine immunization schedule recommended by the WHO. After three immunizations, the Hib conjugate vaccine elicited an immune response, as shown by higher titer of Hib antibody reached 1:8000, 10,666 and 11,733 in serum after one, two and three immunizations in ELISA assay (Fig. 1a). However, this higher titer was identified being similar the concentration of antibody induced in human pediatric clinical in the comparison with human reference serum (Fig. 1a and Additional file 1: Figure S1). The previous serological analysis suggested that the antibody concentration of 0.15 μg/ml in immunized pediatric population was postulated as the positive conversion, and 1 μg/ml as meaning the immunity of long term [47, 48]. The characteristic of the result observed in macaques and been distinct with that usually observed human is that the conjugate vaccine elicited antibody response after 1st immunization and maintained its increasing after 2nd and 3rd immunization [21, 23], as while Hib polysaccharide antigen did weak work in macaques (Fig. 1a). The specific T cell responses were also remarkably induced by Hib conjugate vaccine (Fig. 1b). In vitro, TT could stimulate proliferation of T cells from the monkeys in Hib vaccine group and cytokines secretion (Fig. 1c and d). This result suggests that the Hib conjugate vaccine is capable of eliciting a distinct immune response in 6- to 8-month-old macaques, similar to the effects observed in children older than 18 months of age, whereas the immune response was weak to the polysaccharide antigen. These results were similar to those reported in previous work of capsular polysaccharide-protein conjugates and suggest that rhesus macaques are suitable to use in comparative analyses of the immunological features of Hib [31, 32]. These data also suggest that this model might be limited to reflect the trend of antibody response elicited in the immunized human individuals by Hib CPS vaccine.

### The mRNA gene profile suggested that a synergistic immunologic effect is induced in macaques by the Hib conjugate vaccine compared to CPS and TT

**Fig. 2:**
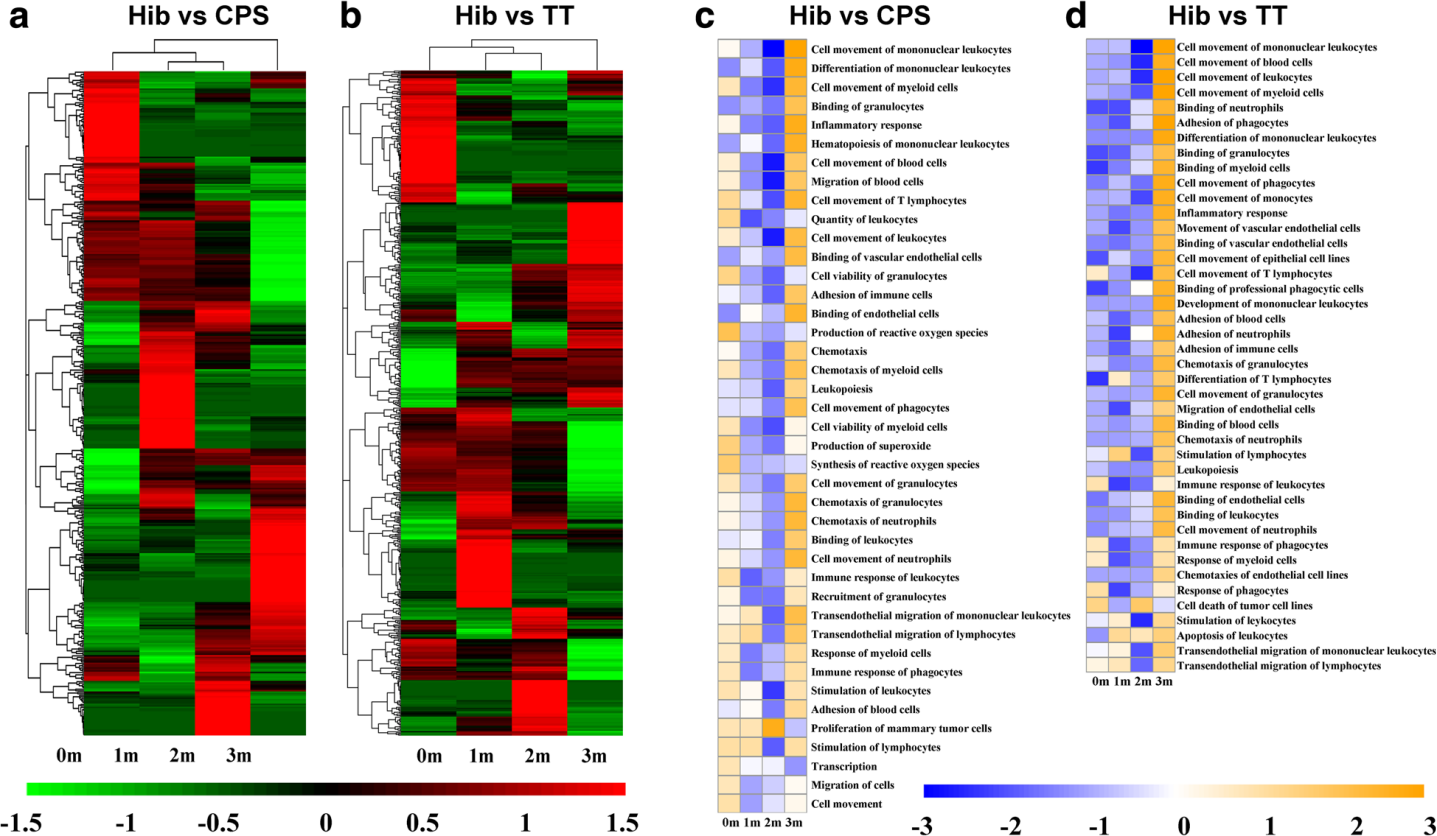
Comparison of mRNA expression in animals immunized with the Hib conjugate vaccine, Hib CPS antigen or carrier TT protein at various time points and IPA analysis of the genes that showed significant differential expression. **a** Comparison of the expression of 437 genes in macaques immunized with the Hib conjugate vaccine or with CPS antigen at various time points. Each row indicates one gene. The values are expressed as log_2_P-values. **b** Comparison of the expression of 596 genes in macaques immunized with the Hib conjugate vaccine or with carrier protein TT at various time points. Each row indicates one gene. The values are expressed as log_2_P-values. **c** IPA analysis of the genes that were differentially expressed in macaques immunized with the Hib conjugate vaccine and in macaques immunized with CPS antigen. The values are expressed as log_2_P-values. **d** IPA analysis of the genes that were differentially expressed in macaques immunized with the Hib conjugate vaccine and in macaques immunized with carrier protein TT. The values are expressed as log_2_P-values

The results of Hib-specific antibody measurements in macaques that were immunized with the Hib conjugate vaccine and Hib capsular antigen or carrier TT protein suggested that the conjugate vaccine, but not the capsular antigen or TT, effectively activated the immune response. This result implied that a synergistic effect was provided by the carrier TT protein and the CPS antigen during conjugate vaccine stimulation of the immune system. To understand the roles of TT carrier protein and CPS antigen in this immune response, we hypothesized that the immune response elicited by Hib conjugate vaccine is involved in a more complicated event for immune system due to the fact that conjugation of CPS and TT might create a changed antigenic structure, although each of TT or CPS could be capable of stimulating immune system [29]. In this case, two comparisons were performed for their mRNA expression profile. A comparison of PBMCs collected from animals immunized with the conjugate vaccine or with CPS suggested that the expression of 437 genes varied significantly in the two groups of animals (Fig. 2a). In the comparison of the gene expression profiles of animals immunized with conjugate vaccine and those of animals immunized with TT protein, 596 genes with significantly different levels of expression were identified (Fig. 2b).

To further understand these differentially expressed genes and their relationship to the immune response to Hib, an Ingenuity Pathway Analysis (IPA) of the genes identified in the comparisons between the Hib conjugate vaccine and polysaccharide antigen groups and between the Hib conjugate vaccine and carrier protein TT groups was performed. The results, which are based on the *P* values of the comparisons between the two immunized animal groups (Fig. 2c, d), yielded two findings. First, the genes with the most significant differences in the conjugate vaccine versus CPS antigen groups were associated with inflammatery response, immune cell adhesionand cell movement of immune cells (Fig. 2c). This result suggests that cellular adhesion and cell movement might have been involved in the production of specific immunity against Hib. Second, the genes with the most significant differential expression in the conjugate vaccine versus the carrier TT group were also associated with cellular adhesion, inflammation and cell movement (Fig. 2d). Taken together, the results from the two comparisons suggested that the major events that occurred during the induction of the Hib immune response may have involved molecules with cellular adhesion functions. In addition, more than 19 genes were associated with cellular adhesion (Additional file 1: Table S3). A previous study showed that the bacterial polysaccharides on the thallus surface, which were as the pathogen associated molecular pattern (PAMP) triggering innate immunity, was capable of interacting with the molecules on human cell surfaces and might play important roles during infection [49], these roles may involve conformational changes in adhesion molecules and subsequent signal transduction and regulation of cellular responses in immune system [50–53]. If these biological processes do occur in immune cells, a functional effect would be involved in the induction of immunity.

### Characterization of genes that appear to be involved in the immune response against Hib in conjugate vaccine-immunized macaques

**Fig. 3:**
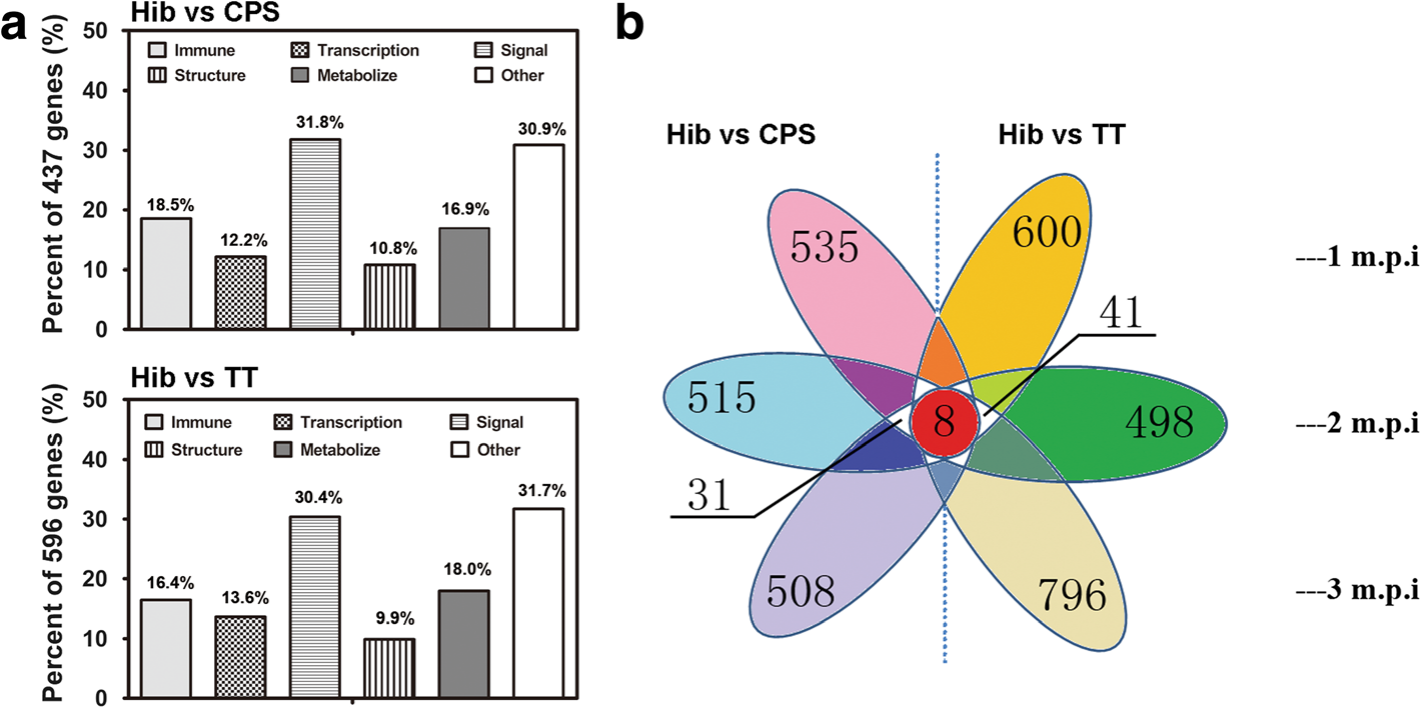
Analysis of the major genes correlated with the antibody response against Hib. **a** Gene classification. The differentially expressed genes (437 in the Hib conjugate vaccine group versus the CPS group and 596 in the Hib conjugate vaccine group versus the TT group) were classified into groups. The figure shows the percent (y-axis) of each clarification of gene (the intersection) to the differentially expressed genes (437 in the Hib conjugate vaccine group versus the CPS group and 596 in the Hib conjugate vaccine group versus the TT group). Multifunctional genes were put into different classes. The percent of differentially classified genes was showed above the column. **b** Global view of the modulation of gene expression in PBMCs obtained from immunized macaques at 1, 2 and 3 months post-inoculation. The individual sets, which are indicated by different colors, show the numbers of significantly modulated genes at each time point. A total of 39 genes (including a specific set of 8 genes) differed between the Hib vaccine and the CPS antigen groups during the entire immunization period. A total of 49 genes (including the same set of 8 genes) differed between the Hib vaccine and the carrier TT groups during the entire immunization period. Thus, a total of 8 genes (shown in red) differed between the vaccine group (Hib vaccine) and the control groups (CPS and TT) during the whole immunization period

The results of IPA analyses of the comparisons of mRNAs derived from cells of animals that received the conjugate vaccine and from those that were immunized with the CPS antigen or carrier protein TT suggested that most of the genes that are involved in the immune response to Hib encode cellular receptors, transcriptional regulators or inflammatory effectors. Further characterization of the differentially expressed genes identified in animals treated with conjugate vaccine versus CPS antigen and conjugate vaccine versus TT protein was performed in accordance with the biological functions of the gene and the time sequence of immunization. The results indicated two findings. First, 437 and 596 differentially expressed genes from the comparisons of conjugate vaccine verse CPS antigen and conjugate vaccine verse TT protein, respectively, were associated with transcription, signal transduction, immune response, cellular structure and metabolism (Fig. 3a); of these, the percentage of genes related to the immune response was 18% and 16% respectively (Fig. 3a). Second, analysis of the expression of individual genes as a function of time after immunization revealed that the differential expression of 31 genes was maintained in the conjugate vaccine versus CPS antigen comparison at the three time points and that differential expression of 41 genes was maintained in the conjugate vaccine versus carrier protein TT comparison at the three time points (Fig. 3b). Examination of the differentially expressed genes in the two groups at the three time points indicated that 8 genes maintained their variations throughout the immunization period (Fig. 3b). Of these 8 genes, five were directly correlated with the induction of the immune response (Table 1), and the other 3 were indirectly associated with it (Table 1). The genes KLRC1, LGALS13 and VNN2 showed higher expression in the animals that were immunized with the conjugate vaccine than in those that were immunized with CPS antigen or with the carrier protein TT. Although it is not clear whether these 8 genes are involved in the immune response, further analysis showed that although KLRC1 expression increased at all three time points in both the CPS antigen and conjugate vaccine groups, its expression remained lower in the CPS antigen group than in the conjugate vaccine group (Table 2). This resulted in larger differences in the comparisons between the two groups. However, LGALS13 expression increased moderately by 3.7-, 1.19- and 9.17-fold in the conjugate vaccine group at the three time points measured, whereas it maintained its decreasing trend in the CPS antigen and carrier protein TT groups. A similar trend was observed for VNN2. This resulted in large differences between the two comparisons (Table 2). These results suggest that there is a logical correlation between the expression of these three genes and the immune response induced by the Hib conjugate vaccine. qRT-PCR analysis of KLRC1, LGALS13, LTB4DH and VNN2 expression in PBMCs of the immunized macaques provided supportive evidence for the results of the other analyses described in this study (Additional file 1: Figure S3).

**Table 1:** The dynamic expressing profile of eight genes associated antibody response in whole immunization period ^a^

	Hib vs Capsular			Hib vs Toxoid		
	1mpi	2mpi	3mpi	1mpi	2mpi	3mpi
KLRC1	1.60	6.51	40.85	2.61	14.72	71.36
LGALS13	5.44	4.76	20.84	17.62	29.75	305.67
LTB4DH	1.40	0.82	1.27	1.36	0.59	0.03
NUAK1	1.70	0.77	2.24	2.58	0.82	2.62
VNN2	0.96	35.12	80.82	0.79	47.70	343.50
GALNT3	0.39	0.48	0.58	1.18	1.66	1.01
LOC710050	0.49	0.75	0.65	0.32	0.53	0.57
LOC716305	1.09	0.76	0.03	0.81	0.69	0.24

^a^ the fold change indicates the expression levels of the specific gene in the samples from Hib vaccine group compared with the samples from Capsular or Toxoid group. The results were normalized to the level of the same gene in the same group at 0 day

**Table 2:** Eight different genes in three groups in whole immunization period ^a^

	Hib			Capsular			Toxoid		
	1mpi	2mpi	3mpi	1mpi	2mpi	3mpi	1mpi	2mpi	3mpi
KLRC1	1.46	6.77	53.52	0.91	1.04	1.31	0.56	0.46	0.75
LGALS13	3.70	1.19	9.17	0.68	0.25	0.44	0.21	0.04	0.03
LTB4DH	1.50	0.67	0.65	1.07	0.82	0.51	1.10	1.14	22.65
NUAK1	2.14	1.19	4.53	1.26	1.55	2.02	0.83	1.46	1.73
VNN2	0.27	31.96	27.48	0.28	0.91	0.34	0.34	0.67	0.08
GALNT3	5.24	2.97	2.32	13.34	6.16	4.00	4.43	1.79	2.29
LOC710050	0.77	0.82	1.05	1.58	1.09	1.61	2.42	1.56	1.83
LOC716305	0.63	0.41	0.18	0.58	0.54	6.08	0.78	0.59	0.74

^a^ the fold change indicates the expression levels of the specific gene in the samples from 1, 2 and 3 m.p.i. compared with the samples from 0 day in the same group

#### Analysis of all characterized genes associated with immunity

**Fig. 4:**
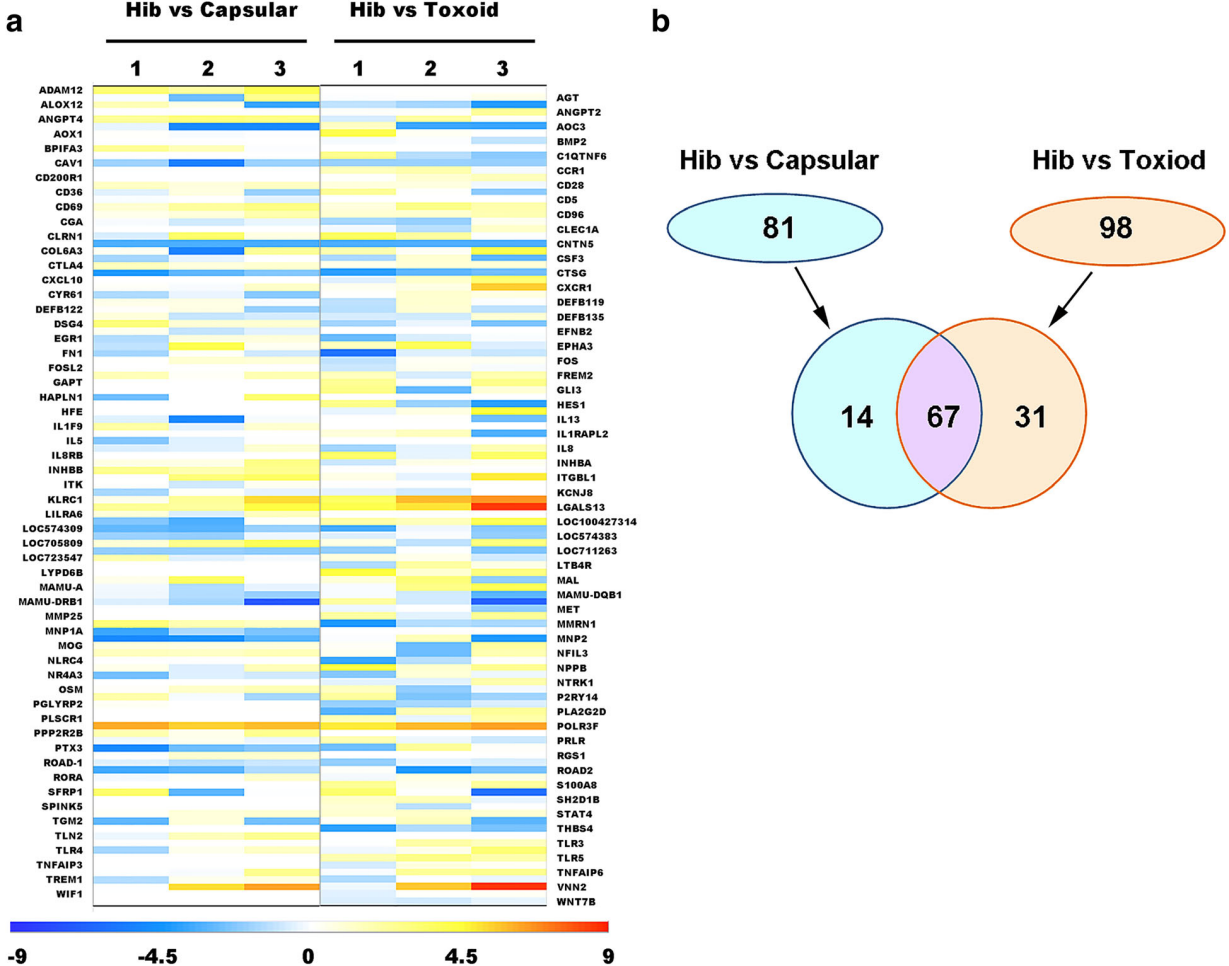
Analysis of the major genes correlated with immunity in Hib group. **a** Immune genes expressions in PBMCs obtained from immunized macaques at 1, 2 and 3 months post-inoculation.The values are expressed as log_2_P-values. **b** Global view of the immune gene expression in PBMCs obtained from immunized macaques at 1, 2 and 3 months post-inoculation. A total of 81 genes (including a specific set of 67 genes) differed between the Hib vaccine and the CPS antigen groups. A total of 98 genes (including the same set of 67 genes) differed between the Hib vaccine and the carrier TT groups. Thus, a total of 67 genes (shown in pink) differed between the vaccine group (Hib vaccine) and the control groups (CPS and TT)

The divergent immune responses and mRNA gene profiles that were induced by immunization with the conjugate vaccine, the CPS antigen and the TT protein suggested that variation in gene expression related to the specific immune response to Hib in immunized macaques might play a role in the function of the immune system. To address this hypothesis, the direction of our investigation next focused on the differentially expressed genes identified in the two comparisons that are likely to function in the immune system. The results of the characterization of the 437 and 596 differentially expressed genes identified the CD28 (cluster of differentiation 28), IL8 (interleukin 8), and TLR4 (Toll-like receptor 4) genes as well as other genes that showed different and varied trends in the two comparisons (Fig. 4a); the latter genes included genes that encode CD (cluster of differentiation) molecules, chemokines, receptors, transcription factors and cytokines (Fig. 4a). It may be speculated that these genes play important roles in the immune response induced by the conjugate vaccine. Furthermore, the up-regulated expression of CD69, a key marker of T cell activation, was observed in Hib conjugated vaccine group compared to the other two groups based on microarray analysis (Fig. 4a); meanwhile, the expression of CD69 in the PBMCs from monkeys immunized by conjugated Hib vaccine group was increased (Additional file 1: Figure S4). Due to the fact that the conjugate vaccine consisted of CPS and TT, the differentially expressed genes identified in the comparison of the conjugate vaccine versus the CPS antigen or the TT protein can be presumed to be complementary. Thus, our work compared the differentially expressed genes observed in a comparison of the conjugate vaccine and TT with the differentially expressed genes observed after three immunizations with CPS alone and after three immunizations with TT alone. Sixty-seven genes were found to be differentially expressed in both comparisons (Fig. 4b), whereas 14 and 31 differentially expressed genes, respectively, were distinctive in each comparison (Fig. 4b). If we postulate that the 67 genes that were differentially expressed in both comparisons are involved in the immunity induced by the conjugate vaccine, the 14 genes may be assumed to be related to the stimulation of the immune system by TT protein, whereas the 31 differentially expressed genes are likely to be those that function in the immune response to CPS antigen. Furthermore, there are 2 genes that are associated with immunity and maintained after three immunizations in both Hib-vs-CPS-comparison groups; and 1 gene that is associated with immunity and maintained after three immunizations in both Hib-vs-TT-comparison groups (Additional file 1: Table S4). Further experimental evidence will be required to support this hypothesis.

## Discussion

Previous studies of various Hib vaccines have suggested that the conjugate vaccine consisting of the CPS antigen and carrier protein is capable of producing strong immunogenicity in immunized populations, although *Haemophilus influenzae type b* capsular polysaccharide could induce a weak immunogenicity [27, 28, 30]. This success has contributed to the development of several bacterial vaccines based on immunological principles and technical criteria [12, 25]. Millions of clinically infectious pediatric Hib cases worldwide have been controlled through the use of the conjugate vaccine over a period of several decades, confirming its efficacy [25]. To study the mechanism of this effective Hib conjugate vaccine-induced immunity, our study compared the mRNA gene profiles of macaques that were immunized with the Hib conjugate vaccine, CPS antigen, or the carrier protein TT and identified specific genes that may play major roles in the induction of the immune response against Hib. Knowledge of these genes can improve our understanding of how immunity is developed against Hib. Interestingly, while a higher immune response occurred in conjugate vaccine-immunized macaques than in those immunized by the CPS antigen or carrier protein TT alone, the genes with the greatest differential expression in the comparisons were associated with cellular adhesion, and these genes could be further classified into genes involved in cellular surface recognition and those involved in interactions between various types of cells in the immune system. This result suggested that the induction of immunity might involve the recognition by immune cells of the conjugated antigen of the polysaccharide and the carrier protein, resulting in subsequent immune cell interactions that activate the adaptive immune response. Several genes whose encoded molecules enabled the induction of the specific immune response and for which dynamic changes in expression were maintained were considered to be significantly correlated with the formation of this immunity. Thus, these genes might play important roles in the immune response. Interesting, the expression of CD69, a key T cell activation marker, was higher in Hib conjugated vaccine group comparing with Capsular and Toxoid groups (Fig. 4a and Additional file 1: Figure S4), supporting that T cells might be activated by Hib vaccine. In addition, unlikely CD69, another gene ITK, which encodes an interleukin 2 induced T cell kinase, was not remarkably changed even down-regulated in Hib vaccine group comparing with the other two groups (Additional file 1: Figure S4 and Fig. 4a).

In the comparisons based upon the time course of response to the conjugate vaccine, CPS and TT, we identified 8 genes that are likely related to the development of immunity; these genes include KLRC1, LGALS13 and VNN2. The encoded product of KLRC1 is NKG2A, an inhibitory receptor that is expressed on the surface of a subset of natural killer (NK) cells and activated CD8^+^ cells [54]. NKG2A controls activation-induced cell death (ACID), which occurs in specifically activated CD8^+^ cells, by forming a heterodimer with CD94 [54]. NKG2A/CD94 maintains immune system homeostasis as anti-infection immunity increases. Interestingly, increasing expression of this gene was maintained from the first to the third month after the first immunization with the Hib conjugate vaccine (1.46- to 53.52-fold relative to the background; Table 2), but little variation in its expression was observed in animals immunized with CPS or TT (0.91 to 1.31-fold and 0.56 to 0.75-fold, respectively; Table 2). This observation suggests that this gene may play an important role in the immune response. Conversely, the LGALS13 gene exhibited a small increasing trend (3.7- to 9.17-fold relative to background; Table 2) during the three-month time period, in distinct contrast to the trends observed in animals immunized with CPS or TT (0.68 to 0.44-fold and 0.21 to 0.03-fold, respectively; Table 2). LGALS13 encodes the PP13 protein, which is classified as a member of the galectin family and is involved in multiple biological processes, including signal transduction and cellular adhesion [55–60]. PP13 also induces the secretion of inflammatory agents and chemokines by mononuclear cells [61, 62]. A third gene, VNN2, exhibited conjugate vaccine-induced variation (0.27 to 27.48-fold; Table 2) that was distinctly different from the variations induced by vaccination with CPS or TT alone (0.28 to 0.34-fold and 0.34 to 0.08-fold; Table 2). VNN2 encodes a glycosyl phosphatidylinositol (GPI)-anchored protein that regulates cellular adhesion and promotes neutrophil migration and inflammatory reactivity [63–65].

In addition to the above, we classified the differentially expressed genes identified in various comparisons based upon immunological characterization and tested the hypothesis that the genes whose expression is specifically induced by the conjugate vaccine and not by CPS represent the difference in the integral immunity induced by an intact immunogen and the deficient immunity induced by a hapten antigen. Similarly, the genes that were specifically induced by the conjugate vaccine and not by TT could be postulated to reflect the difference in integral immunity induced by an intact immunogen compared to the deficient immunity induced by a carrier protein. In this case, the 17 differentially expressed genes observed in comparisons between conjugate vaccine and TT and CPS alone (Fig. 4b) could help explain the carrier’s role in the resulting immunity. Among these genes, we identified CCR1, CXCL10 and CXCR1, which are involved in inflammation [66], as well as EGR1, NLRC1, NTRK1 and MET, which mediate the interaction of immune cells and signal transduction in the immune system [67–70]. The identification of these genes suggests a possible mechanism for the role of the carrier protein in the immune response. On the other hand, the 67 different genes identified in the comparison between conjugate vaccine and CPS or TT alone (Fig. 4b) may offer clues regarding the role of CPS in immunity; for example, IL5, a cytokine that stimulates the differentiation and growth of B cells [71], showed up-regulation in animals immunized with the conjugate vaccine and down-regulation in the animals immunized with CPS or TT only (Fig. 4b). This finding suggests that IL5 played a key role in the immunity. In contrast, the OSM gene, which has been shown to modulate the expression of IL6, GM-CSF and G-CSF [72, 73] was not induced in animals immunized with the conjugate vaccine, whereas its expression increased in animals that were immunized with CPS or TT only (Fig. 4b). This result also implies a key role of the OSM gene in immunity to Hib.

Despite that we’re only allowed to perform the experiments on limited number of animals due to the funding restriction, we still noticed a remarkable immune response in vaccine immunization group. Based on our analyses, we propose that the combination of the Hib polysaccharide antigen and the carrier protein TT produces an antigenic structure that is capable of activating both innate and adaptive immune responses. This Hib conjugate vaccine might achieve this effect through its recognition by the molecules in the surface of some immune cells followed by activation of innate and adaptive immune responses which is characterized by an increase in the level of antibodies against Hib and by the maintenance of that level. The CPS antigen is unable to induce an effective antibody response probably with its lacking the appropriate structure. However, our model will require experimental validation in specific animal models with deficient genes.

## Conclusions

Probably as a result of the synergistic effects between the carrier TT and CPS antigen in Hib conjugate vaccine, several special genes associated with specific immunity in the Hib antibody induction and maintenance, were primarily induced.

## Additional file

Additional file 1: Table S1. two monkeys in each group were immunized intramuscular injection. **Table S2.** The primers of eight genes for real-time RT-PCR. **Figure S1** ELISA analysis of total antibody titers from immunized rhesus macaques. **Figure S2.** ELISA analysis of antibody from immunized rhesus macaques. **Figure S3.** qRT-PCR analysis of mRNA at various time points. **Figure S4.** qRT-PCR and Flow cytometry analysis of CD69 and ITK expression. **Tables S1**, **S2**, **S3**, **S4**, **Figures S1**, **S2**, **S3** and **S4.** A remarkable immune response induced by Hib conjugate vaccine, in rhesus macaques, because of a result of the synergistic effects between the carrier TT and CPS antigen. (DOCX 1352 kb)

## Abbreviations

CD: Cluster of differentiation
CD28: Cluster of differentiation 28
CD69: Cluster of differentiation 69
CPS: Capsular polysaccharide
DT: Diphtheria toxoid
Hib: *Haemophilus influenzae* type b
IL8: Interleukin 8
IPA: Ingenuity Pathway Analysis
ITK: IL2 inducible T cell kinase
OMP: Outer membrane protein
PBMCs: Peripheral blood mononuclear cells
PRP: Polyribosylribitol phosphate
RT: Room temperature
TLR4: Toll-like receptor 4
TT: Tetanus toxoid
