# Supplementary material for: RNA gene profile variation in peripheral blood mononuclear cells from rhesus macaques immunized with Hib conjugate vaccine, Hib capsular polysaccharide and TT carrier protein
Source: BMC Immunol. 2018 Jan 25;19:4. doi: 10.1186/s12865-018-0240-5 (PMC5784715; doi:10.1186/s12865-018-0240-5)
Supplement: Additional file 1: Table S1. — two monkeys in each group were immunized intramuscular injection. Table S2. The primers of eight genes for real-time RT-PCR. Figure S1 ELISA analysis of total antibody titers from immunized rhesus macaques. Figure S2. ELISA analysis of antibody from immunized rhesus macaques. Figure S3. qRT-PCR analysis of mRNA at various time points. Figure S4. qRT-PCR and Flow cytometry analysis of CD69 and ITK expression. Tables S1, S2, S3, S4, Figures S1, S2, S3 and S4. A remarkable immune response induced by Hib conjugate vaccine, in rhesus macaques, because of a result of the synergistic effects between the carrier TT and CPS antigen. (DOCX 1352 kb) [file 12865_2018_240_MOESM1_ESM.docx]

**Additional file**

**Table S1** Two monkeys in each group were immunized intramuscular injection

| **Group** | **Antigen preparation** | **Description** | **Volume** |
| --- | --- | --- | --- |
| 1 | Act-HIB | 10 μg CPS+30 μg TT | 0.5 ml |
| 2 | Tetanus toxoid | 30 μg TT | 0.5 ml |
| 3 | Polysaccharide | 10 μg CPS | 0.5 ml |

**Table S2** The primers of eight genes for real-time RT-PCR.

| **Gene name** | **Primer sequence (5’→3’)** | |
| --- | --- | --- |
|  | **Sense** | **Anti-sense** |
| KLRC1 | CTCCTCACCCACATCCACTC | CTGTTTCCCTACACCCAACCT |
| LGALS13 | GGGAGATGTTGGTCAAAGGA | AGGCAGTTTGTATGGCACCT |
| LTB4DH | GGGAGATGTTGGTCAAAGGA | AACGAAGGAGGAGGAAGAGC |
| NUAK1 | GGGTTTAGGTTGGCTGGAAT | CTCGCTCACCATTGCTCATA |
| VNN2 | GTCACTCTTTGGGAGGTGGT | ACTTGGGAAGCCGATAAACA |
| GALNT3 | TTTCCCTCCCTCTCCCTCTA | CTGTTACCTGCTTGGGCTGT |
| LOC710050 | TGTGGTGCGGTGGTAGATTA | TATCCAGTCCCGGTATCAGC |
| LOC716305 | TCCTGCCTTTCTGAGGTTGT | CTTTGCCCACCAAGTTCCTA |
| CD69 | GGTACCTCCCCTCATCCTCA | CCACTCAGAAACAGAAGGAAACC |
| ITK | CGTTTCAGGTGGTGCATGAC | ACACGGTGTATGTTCCTGCA |

**Table S3** The significant different genes associated with cell adhesion comparing between the Hib vaccine and CPS group and between the Hib vaccine and TT group.

| **Name (from A to Z)** |  | **Hib vaccine vs CPS group** | | |  | **Hib vaccine vs TT group** | | |
| --- | --- | --- | --- | --- | --- | --- | --- | --- |
|  |  | 1 | 2 | 3 |  | 1 | 2 | 3 |
| ADAM12 |  | 9.51 | 4.69 | 16.37 |  | 12.92 | 11.69 | 18.59 |
| AOC3 |  | 0.75 | 0.82 | 0.03 |  | 4.13 | 1.99 | 0.07 |
| ARVCF |  | / | / | / |  | 0.75 | 0.97 | 0.07 |
| BMX |  | / | / | / |  | 1.22 | 0.97 | 23.83 |
| BOC |  | / | / | / |  | 2.84 | 1.14 | 1.56 |
| CCR1 |  | / | / | / |  | 0.62 | 0.61 | 0.53 |
| CD36 |  | 0.68 | 1.62 | 0.25 |  | 0.89 | 1.05 | 0.22 |
| CD96 |  | 1.36 | 1.77 | 3.74 |  | 2.05 | 2.13 | 3.37 |
| CNTN2 |  | / | / | / |  | 13.96 | 1.08 | 3.02 |
| CNTN5 |  | 0.09 | 0.09 | 0.09 |  | 0.05 | 0.10 | 0.09 |
| COL4A6 |  | / | / | / |  | 0.16 | 0.44 | 0.28 |
| COL6A3 |  | 1.22 | 6.05 | 5.35 |  | 0.97 | 1.16 | 16.80 |
| COL8A1 |  | / | / | / |  | 4.49 | 0.80 | 1.88 |
| DSG4 |  | 8.49 | 1.91 | 2.07 |  | 4.59 | 0.76 | 0.18 |
| EFNB2 |  | 1.32 | 0.46 | 0.68 |  | / | / | / |
| EPHA3 |  | 0.43 | 17.78 | 1.24 |  | 0.73 | 14.33 | 0.64 |
| F8 |  | / | / | / |  | 0.55 | 0.63 | 0.43 |
| FAT3 |  | / | / | / |  | 4.14 | 1.20 | 1.27 |
| FN1 |  | 0.30 | 1.13 | 0.54 |  | 0.55 | 0.65 | 0.49 |
| FREM2 |  | 3.07 | 1.14 | 3.38 |  | 3.39 | 0.61 | 3.68 |
| HAPLN1 |  | 0.15 | 1.00 | 10.07 |  | 0.92 | 0.96 | 3.27 |
| HAS1 |  | 1.35 | 0.47 | 2.12 |  | 0.45 | 0.15 | 2.94 |
| HES1 |  | / | / | / |  | 0.21 | 0.27 | 0.06 |
| ITGBL1 |  | 1.00 | 8.73 | 12.39 |  | 1.50 | 11.46 | 29.20 |
| MAGI1 |  | / | / | / |  | 0.97 | 2.66 | 1.16 |
| MMRN1 |  | 8.40 | 3.36 | 0.76 |  | 2.51 | 2.84 | 0.33 |
| MOG |  | 1.74 | 1.60 | 1.70 |  | 1.75 | 1.19 | 4.55 |
| PCDHB3 |  | 0.30 | 0.18 | 0.16 |  | 0.18 | 0.18 | 0.16 |
| SIGLEC5 |  | 1.47 | 0.84 | 1.56 |  | / | / | / |
| SPON1 |  | 1.62 | 1.11 | 0.54 |  | 0.96 | 1.62 | 0.38 |
| THBS4 |  | / | / | / |  | 0.77 | 0.45 | 0.17 |
| TLN2 |  | 0.79 | 2.90 | 5.78 |  | 1.30 | 34.61 | 6.66 |
| TNFAIP6 |  | 0.98 | 0.93 | 6.08 |  | 0.92 | 0.90 | 6.11 |

/: un-changed or NA

**Table S4.** The significant differentially expressed genes that are both associated with immunity and maintained after three immunizations in both Hib-vs-CPS-comparison groups and in both Hib-vs-TT-comparison groups.

| **Name** |  | **Hib-vs-CPS** | | | | | | |  | **Hib-vs-TT** | | | | | | |
| --- | --- | --- | --- | --- | --- | --- | --- | --- | --- | --- | --- | --- | --- | --- | --- | --- |
|  |  | 31 different genes | | |  | 81 different genes | | |  | 41 different genes | | |  | 98 different genes | | |
|  |  | 1 | 2 | 3 |  | 1 | 2 | 3 |  | 1 | 2 | 3 |  | 1 | 2 | 3 |
| ALOX12 |  | 3.12 | 1.47 | 0.07 |  | 7.32 | 3.44 | 0.17 |  | / | / | / |  | / | / | / |
| MAMU-A |  | 0.86 | 0.37 | 0.73 |  | 0.29 | 0.12 | 0.03 |  | / | / | / |  | / | / | / |
| IL8 |  | / | / | / |  | / | / | / |  | 0.30 | 0.72 | 3.34 |  | 0.25 | 0.29 | 3.88 |


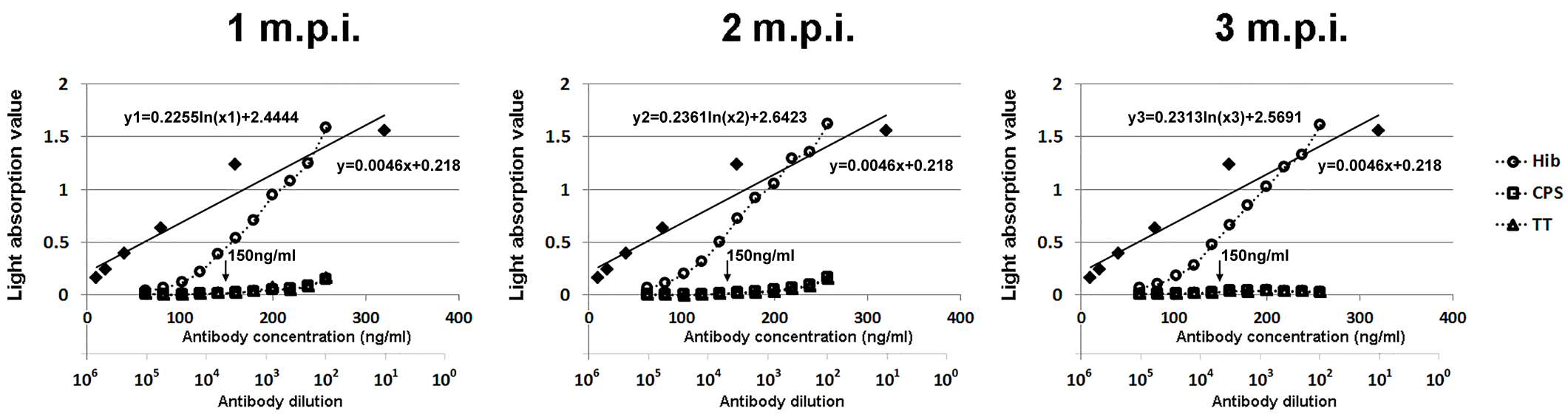


**Figure S1 ELISA analysis of total antibody titers from immunized rhesus macaques**

Diluted macaque serum was showed by antibody dilution (x-axis, below). Antibody concentration of standard human serum against Hib (“diamond” in figure) was used as reference control (x-axis, up) and drawn a straight line (trend line). The antibody concentration (0.15 μg/ml) to protect from bacteremic infection was showed by arrow. Y-axis is the light absorbance value in wave length of 450 nm. Antibody concentration of standard human serum was calculated by the formula of y=0.0046x+0.218. And monkey antibody dilution of Hib vaccine group was calculated by the formula of y1, y2 and y3 respectively. Each point is average means of the results of three independent experiments.

**
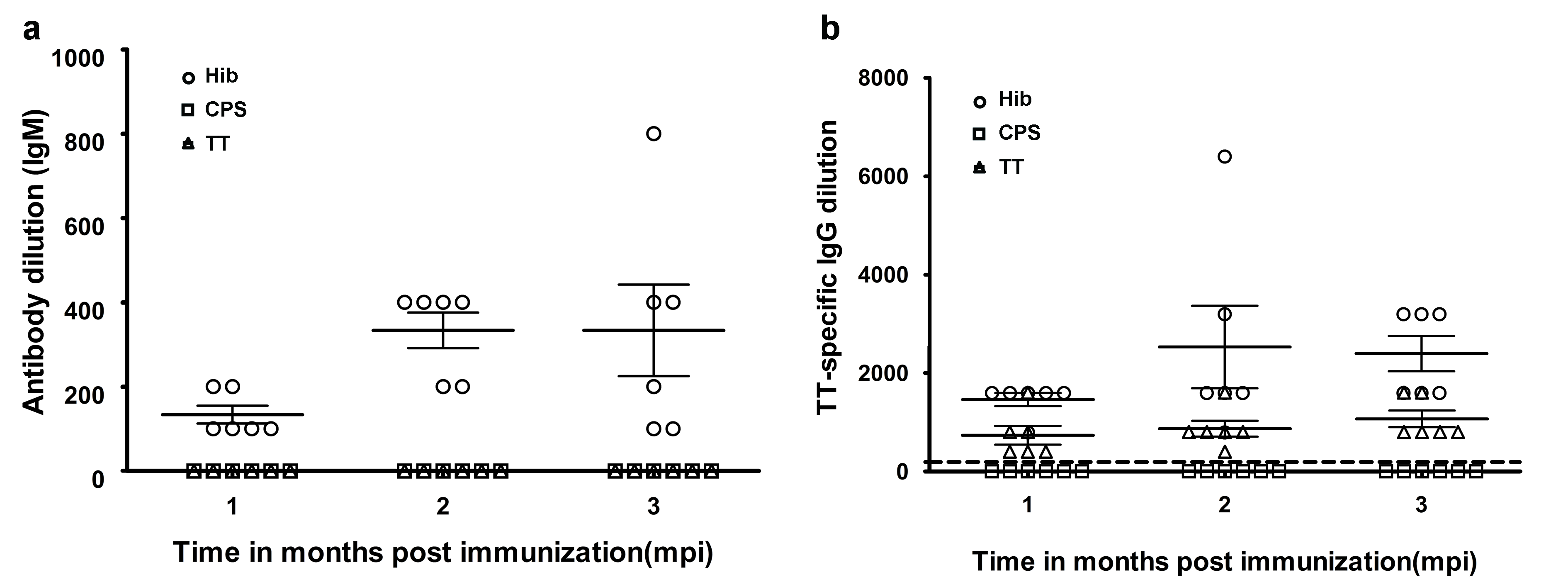
**

**Figure S2 ELISA analysis of antibody from immunized rhesus macaques**

1. The polysaccharide-specific IgM induced by Hib conjugate vaccine, TT and CPS.
2. The carrier protein-specific IgG induced by Hib conjugate vaccine, TT and CPS. The dotted line indicates the TT-specific IgG dilution value of positive (in this dilution the titer of IgG=0.11U/ml).


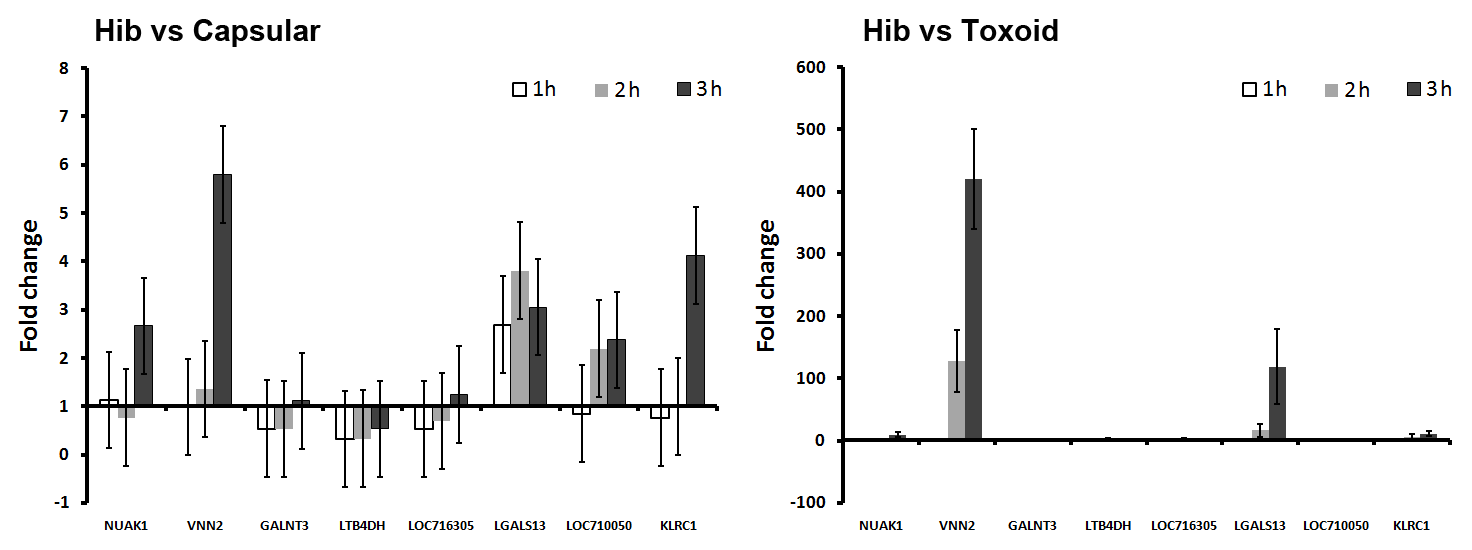


**Figure S3 qRT-PCR analysis of mRNA at various time points**

The results were normalized to endogenous GAPDH. The y-axis indicates the relative quantity of the specific mRNA in the samples compared with the control. The error bars indicate the SD of the relative quantities.


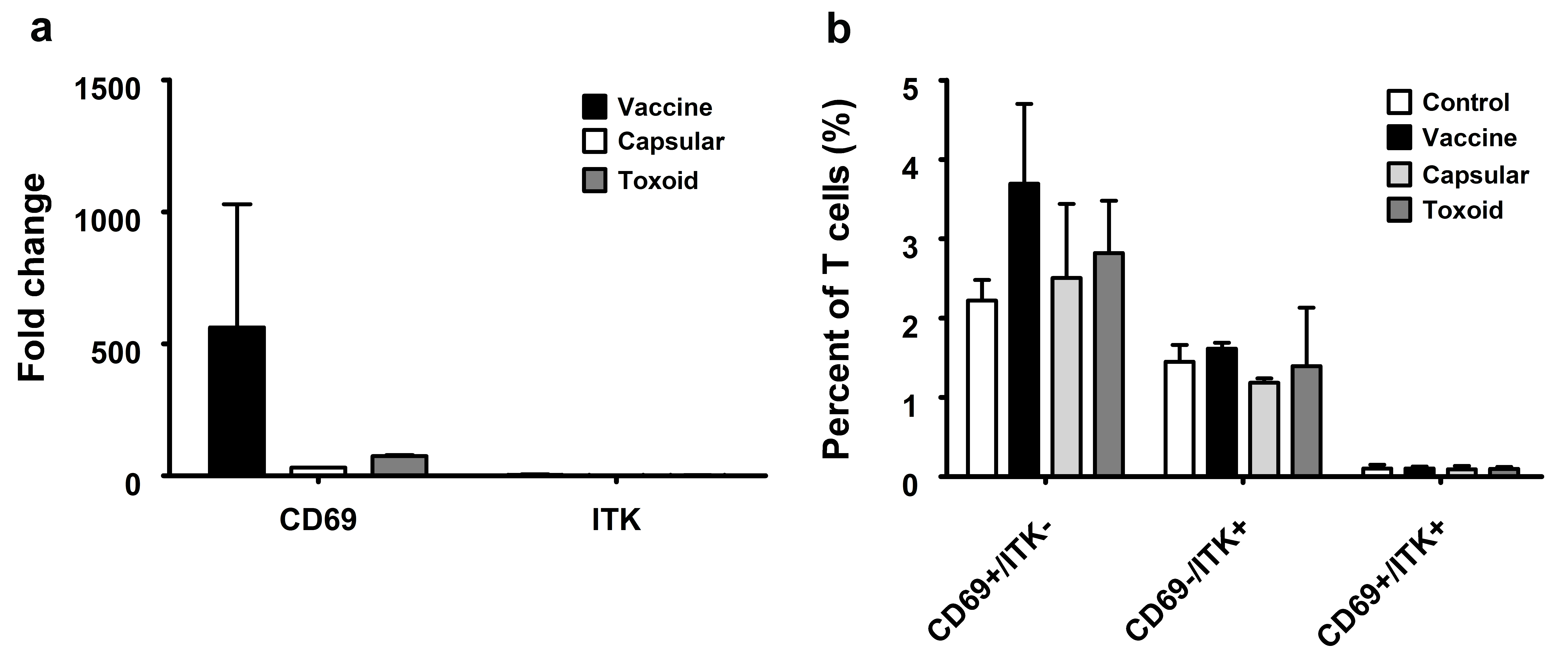


**Figure S4 qRT-PCR and Flow cytometry analysis of CD69 and ITK expression**

1. The total RNAs from PBMCs of immunized monkeys were extracted. The results were normalized to endogenous GAPDH. The y-axis indicates the relative quantity of the specific mRNA in the samples compared with the control.
2. PBMCs of immunized monkeys from each group were stained and analyzed by flow cytometry. T cells were gated by CD3 and ten thousand cells were used for analysis.
